# Geometric structure of spinal curves: application to adolescent idiopathic scoliosis

**DOI:** 10.1186/1748-7161-8-S1-P12

**Published:** 2013-06-03

**Authors:** J Deceuninck, Jc Bernard, E Berthonnaud

**Affiliations:** 1 Croix Rouge française CMCR Les Massues, Lyon, France; 2 L’Hôpital Nord Ouest, Villefranche sur Saône, France; 3 Laboratoire de Physiologie de l’Exercice, Université de Lyon, Saint Etienne, France

## Background

The spinal pattern of asymptomatic subjects is generally described using sagittal radiographic images. Frontal radiographic exposures allow clinical people to access to spinal deformations due to scoliosis. Biplanar radiographic examination, coupled with photogrammetric reconstructions, may be used for reconstructing the 3D spinal curve.

## Aim

This communication presents a new study of the geometric structure of 3D spinal curves in adolescent idiopathic scoliosis.

## Discussion

The spine is considered as an heterogeneous beam, and is modeled as a deformable wire, along which vertebrae are beads rotating about the wire. Each vertebra can rotate about the 3D spinal curve. 3D spinal curves are compound of plane regions connected together by zones of transition. The 3D spinal curve is uniquely flexed along the plane regions. The angular offsets between adjacent regions are concentrated at the level of the middle zones of transition. The plane regions along the 3D spinal curve must satisfy two criteria: i) a criterion of minimum distance between the curve and the regional plane and ii) a criterion controlling that the curve is continuously plane at the level of the region.

## Conclusion

The geometric structure of spinal curve is characterized by the sizes, and functions, of zones of transition. Spinal curves of asymptomatic subjects show three plane regions corresponding to spinal curves. In some scoliotic spines, four plane regions may be detected, and zones of transition may be lengthened, and not at the same place.
